# The pro-radical hydrogen peroxide as a stable hydroxyl radical distributor: lessons from pancreatic beta cells

**DOI:** 10.1007/s00204-022-03282-6

**Published:** 2022-04-13

**Authors:** Sigurd Lenzen, Volodymyr I. Lushchak, Fritz Scholz

**Affiliations:** 1 Institute of Experimental Diabetes Research, Hannover Medical School, 30625 Hannover, Germany; 2 Institute of Clinical Biochemistry, Hannover Medical School, Hannover, Germany; 3 Department of Biochemistry and Biotechnology, Vasyl Stefanyk Precarpathian National University, Ivano-Frankivsk, Ukraine; 4 I. Horbachevsky Ternopil National Medical University, Ternopil, Ukraine; 5 Institute of Biochemistry, University of Greifswald, Greifswald, Germany

**Keywords:** Oxidative stress, Hydrogen peroxide, Hydroxyl radical, Pancreatic beta cell

## Abstract

The toxic potential of H_2_O_2_ is limited, even if intracellular concentrations of H_2_O_2_ under conditions of oxidative stress increase to the micromolar concentration range. Its toxicity is mostly restricted to the oxidation of highly reactive thiol groups, some of which are functionally very important. Subsequently, the HO^·^ radical is generated spontaneously from H_2_O_2_ in the Fenton reaction. The HO^·^ radical is extremely toxic and destroys any biological structure. Due to the high reactivity, its action is limited to a locally restricted site of its generation. On the other hand, H_2_O_2_ with its stability and long half-life can reach virtually any site and distribute its toxic effect all over the cell. Thereby HO^·^, in spite of its ultra-short half-life (10^–9^ s), can execute its extraordinary toxic action at any target of the cell. In this oxidative stress scenario, H_2_O_2_ is the pro-radical, that spreads the toxic action of the HO^·^ radical. It is the longevity of the H_2_O_2_ molecule allowing it to distribute its toxic action from the site of origin all over the cell and may even mediate intercellular communication. Thus, H_2_O_2_ acts as a spreader by transporting it to sites where the extremely short-lived toxic HO^·^ radical can arise in the presence of “free iron”. H_2_O_2_ and HO^·^ act in concert due to their different complementary chemical properties. They are dependent upon each other while executing the toxic effects in oxidative stress under diabetic metabolic conditions in particular in the highly vulnerable pancreatic beta cell, which in contrast to many other cell types is so badly protected against oxidative stress due to its extremely low H_2_O_2_ inactivating enzyme capacity.

## Introduction

In the process of oxidative phosphorylation, energy is harvested as ATP via a process of reduction of a dioxygen molecule to two water molecules. ROS (reactive oxygen species) are products of incomplete reduction of molecular O_2_ (oxygen). H_2_O_2_ (hydrogen peroxide) as the 2-electron reduction product of O_2_ is not fully reduced and more reactive than dioxygen. At variance, the O_2_^·−^ (superoxide radical anion) and the HO^·^ (hydroxyl radical) are 1-electron reduction products of O_2_ and of H_2_O_2_, respectively [for details, see Lenzen (2017)]:

$${\text{O}}_{2} \xrightarrow{{{\rm e}^{ - } }} {\text{O}}_{2}^{ \cdot - } \xrightarrow[{{2{\text{H}}^{ + }}}]{{\text{e}}^{ - }} {\text{H}}_{2} {\text{O}}_{2} \xrightarrow{{{\rm e}^{ - } }} {\text{HO}}^{ \cdot } {\text{ + HO}}^{ - } \xrightarrow[{{2{\text{H}}^{ + }}}]{{\text{e}}^{ - }} {\text{2H}}_{2} {\text{O}}{}$$

Although O_2_ has the same oxidation state in HO^·^ and H_2_O_2_, the standard potential of the HO^·^ radicals is exceedingly higher:

$$\begin{aligned} {\text{O}}_{{2 }} + {\text{2H}}^{ + } + {\text{2e}}^{ - } \, \leftrightarrows {\text{H}}_{{2}} {\text{O}}_{{2}} \quad E_{{ {\text{O}}_{{2}} {\text{/H}}_{{2}} {\text{O}}_{{2}} }}^{{\rlap{--} \bigcirc -}} \, = \, 0.695{\text{ V vs. SHE,}}  \\ {\text{H}}_{{2}} {\text{O}}_{{2}} + {\text{2H}}^{ + } + {\text{2e}}^{ - }  \leftrightarrows {\text{2H}}_{{2}} {\text{O}}\quad E_{{ {\text{H}}_{{2}} {\text{O}}_{{2}} {\text{/H}}_{{2}} {\text{O}} }}^{{\rlap{--} \bigcirc- }} = \, 1.763{\text{ V vs. SHE,}}  \\ {\text{O}}_{{2 }} + {\text{ 4H}}^{ + } {\text{ + 4e}}^{ - } \, \leftrightarrows {\text{ 2H}}_{{2}} {\text{O }} \quad E_{{{\text{O}}_{{2}} {\text{/H}}_{{2}} {\text{O}}}}^{{\rlap{--} \bigcirc -}} \, = \, 1.229{\text{ V vs. SHE,}}  \\ {\text{H}}_{{2}} {\text{O}}_{{2}} + {\text{ H}}^{ + } {\text{ + e}}^{ - }  \leftrightarrows {\text{ HO}}^{ \cdot } {\text{ + H}}_{{2}} {\text{O }} \quad E_{{ {\text{H}}_{{2}} {\text{O}}_{{2}} {\text{ HO}}^{ \cdot } }}^{{\rlap{--} \bigcirc- }} = { 0}.714{\text{ V vs. SHE,}}\\ {\text{HO}}^{ \cdot } + {\text{ H}}^{ + } {\text{ + e}}^{ - } \, \overset {} \leftrightarrows {\text{ H}}_{{2}} {\text{O }} \quad E_{{{{\text{ HO}}^{ \cdot }\text{/H}}_{{2}} {\text{O}}}}^{{\rlap{--} \bigcirc- }} \, = \, 2.813{\text{ V vs. SHE}}{.} \end{aligned}$$

This is so because of the stoichiometry and the fact that not the standard potentials, but the free energies are additive (Luther’s rule) (Scholz 2012):

$$\begin{aligned} {\Delta }G_{{{\text{H}}_{{2}} {\text{O}}_{{2}} /{\text{HO}}^{ \cdot } }}^{{\rlap{--} \bigcirc- }} + {\Delta }G_{{{\text{HO}}^{ \cdot } {\text{/H}}_{{2}} {\text{O}}}}^{{\rlap{--} \bigcirc- }} & = {\Delta }G_{{{\text{H}}_{{2}} {\text{O}}_{{2}} {\text{/H}}_{{2}} {\text{O}}}}^{{\rlap{--} \bigcirc- }} \\ {\Delta }G_{{\text{i/j}}}^{{\rlap{--} \bigcirc- }} & = - n_{{\text{i/j}}} FE_{{\text{i/j}}}^{{\rlap{--} \bigcirc- }} \\ FE_{{{\text{H}}_{{2}} {\text{O}}_{{2}} /{\text{HO}}^{ \cdot } }}^{{\rlap{--} \bigcirc- }} + FE_{{{\text{HO}}^{ \cdot } {\text{/H}}_{{2}} {\text{O}}}}^{{\rlap{--} \bigcirc -}} \, & { = 2}FE_{{{\text{H}}_{{2}} {\text{O}}_{{2}} {\text{/H}}_{{2}} {\text{O}}}}^{{\rlap{--} \bigcirc- }} \, {} \\ \end{aligned}$$

Of the partially reduced O_2_ products, only H_2_O_2_ is kinetically sufficiently stable, a small electroneutral molecule in its non-dissociated form (p*K*_a_, 11.75), and endowed with chemical properties, which allow this reactive species to travel long distances and cross biological membranes due to which it can reach reaction sites far away from the site of its generation. These are physicochemical features which put H_2_O_2_ into a crucial role, when large amounts of H_2_O_2_ (in the micromolar concentration range) are generated under conditions of oxidative stress (Halliwell 2006; Halliwell and Gutteridge 2015; Sies 1986, 2014; Winterbourn 2008; Winterbourn 2013).

H_2_O_2_ is the intermediate reduction product of O_2_, generated in the peroxisomes and the ER (Lenzen 2017), while it is the reduction product of O_2_^·−^ in mitochondria and the cytosol, where H_2_O_2_ is generated in an initial step by the SOD isoenzymes MnSOD and Cu/ZnSOD or spontaneously (Lenzen 2008; Lortz et al. 2005; Lushchak 2014; Winterbourn 2020). Thus, at all subcellular sites, H_2_O_2_ is ultimately one of the ROS of crucial importance as a mediator of toxicity under conditions of oxidative stress (Jones and Sies 2015; Lenzen 2017; Lushchak 2014).

We will present here a new interpretation of the role of H_2_O_2_ in the mediation of oxidative stress-induced dysfunction and death in the very vulnerable (Lenzen 2008 #34) and badly protected pancreatic beta cell (Lenzen 2017), when compared to many other cell types (Lenzen et al. 1996; Tiedge et al. 1997) in the development of a diabetic metabolic state. We explain that H_2_O_2_ is not only a reactive species with toxic potential locally at the site of H_2_O_2_ generation but also spreads because of its physicochemical properties the toxicity throughout the whole cell, thereby spreading the toxicity of the HO^·^ radical ultimately generated in the Fenton reaction from H_2_O_2._

## The central role of hydrogen peroxide as a pro-radical in the mitochondria and the peroxisomes

H_2_O_2_ can traverse lipid membranes (Lushchak 2011), in particular through some members of the aquaporin family, the so-called peroxiporins by facilitated diffusion (Bienert and Chaumont 2014; Laporte et al. 2020; Lenzen 2017), thereby entering the cytosolic compartment irrespective of the subcellular site of its generation, may it be the mitochondria or the peroxisomes. The number of fast reacting high affinity sites, namely the thiol (sulfhydryl, SH) groups of the antioxidative enzymes of the glutathione peroxidase (GPx) and peroxiredoxin (Prx) enzyme families is small due to the low expression levels in the mitochondria and the peroxisomes of the pancreatic beta cells (Lenzen 2017). Therefore, only little of the high amounts of H_2_O_2_ generated in these organelles during cytokine toxicity in the T1DM scenario (Lenzen 2017) in the mitochondria and glucolipotoxicity in the T2DM scenario (Gehrmann et al. 2010; Lenzen 2017) in the peroxisomes are quickly inactivated at the sites of H_2_O_2_ generation. These are optimal prerequisites for longevity and long-distance travel of H_2_O_2_. This allows a distribution of H_2_O_2_ over the entire organelle of origin and beyond in the cytosolic space across the surrounding lipid membranes through facilitated diffusion via peroxiporins. Under these conditions, H_2_O_2_ can travel as long and as far until it meets free iron (II) or copper (I) ions or other suitable electron donors (Lenzen 2017). Instead of an interaction of H_2_O_2_ with a protective high affinity thiol, it can hit such a free ion, which acts as a catalyst for the formation of the most highly toxic hydroxyl radical (HO^·^) (Lenzen 2017). The high toxicity of the HO^·^ radical has thermodynamic and kinetic reasons. Its very high standard oxidative potential provides the thermodynamic driving force for many oxidations, and its small radius and uncharged state provides a great mobility, thereby allowing high rates of chemical reactions.

## Relation between the subcellular organelles in the generation of hydrogen peroxide and the hydroxyl radical in the pancreatic beta cell

The volume of the beta cell covered by the mitochondria and the peroxisomes is very low, 4% and less than 1% of the cytosolic ground substance, respectively (Dean 1973; Lenzen and Panten 1983). The cytosolic ground substance comprises more than 50% of the beta cell volume (Dean 1973). The consequence of the very low beta cell mitochondrial volume and the even smaller peroxisomal volume is that any crossing of H_2_O_2_ into the surrounding cytoplasmic space results in a decrease of the H_2_O_2_ concentration in these organelles through the dilution in a larger cellular space. This can result in a lower H_2_O_2_ toxicity in the organelle of origin. Vice versa, it is not surprising that a knockout of a peroxiporin (e.g., aquaporin-8) can result in an increased H_2_O_2_ toxicity in the mitochondria through an increased steady-state H_2_O_2_ level after exposure of the insulin-producing cells to proinflammatory cytokines (Lortz et al. 2005). On the other hand, this widespread distribution over the whole cytosolic space may allow H_2_O_2_ to reach many different intracellular sites. However, the ultimate reason for the toxicity of H_2_O_2_ and the pronounced vulnerability of the pancreatic beta cell is the low level expression of H_2_O_2_-eliminating enzymes (Lenzen 2008, 2017, 2021). This explains the long persistence of the H_2_O_2_ molecule in the beta cell and maximizes the chance to be converted to the highly toxic HO^·^ at sites all over the beta cell, in particular when H_2_O_2_ meets weakly complexed iron (II) ions (Halliwell and Gutteridge 2015; Lenzen 2017; Winterbourn 2013). It has been speculated that HO^·^ radicals may follow a diffusion mechanism, which has a similarity with the famous Grotthuss mechanism (Osakai 2012) of H^+^ and HO^−^ diffusion (so-called proton tunneling). Of course, in the case of the HO^·^ radicals a hydrogen atom abstraction should occur instead of a proton abstraction (in fact, only bonds are moving). Very recently, it has been shown that HO^·^ radicals do not diffuse via a hydrogen atom abstraction (Vassilev et al. 2005). Rather the diffusion rate of the HO^·^ radical (like that of H^+^ and HO^−^ ions) is typical for its physical size. An exceptionally high diffusion rate would also contribute to a higher reaction rate with whatever targets. Thus, the high reactivity of the HO^·^ radical, which also extends to the noble metals (Au, Pt, Pd, Ag) (Nowicka et al. 2010a, b, 2011), has to be solely attributed to its radical nature.

Thus, H_2_O_2_ plays the role of a pro-radical in the weakly protected beta cell (Lenzen 2008). That means that virtually any protein, carbohydrate, lipid or nucleic acid is a potential target for the toxic action of the highly toxic HO^·^ radical with its extremely high reactivity (Lenzen 2017; Lushchak 2014). This explains the universal unspecific toxicity of the HO^·^ radical towards any chemical structure at the site of its generation against which no protection whatsoever is possible. A protection is possible only by elimination of H_2_O_2_, which, however, is limited in the pancreatic beta cell due to the low level of expression of H_2_O_2_-eliminating enzymes (Lenzen 2017).

## The role of the superoxide radical anion

When the primary reactive oxygen species generated is not H_2_O_2_ but the O_2_^·−^ radical anion, O_2_^·−^ is efficiently transformed into H_2_O_2_ through SOD isoenzymes (Lortz et al. 2005). This is in particular true for the highly expressed MnSOD in the beta cell mitochondria (Lortz et al. 2005) so that also when O_2_^·−^ is initially generated, it is instantaneously available as H_2_O_2_ owing to the action of SOD (Mehmeti et al. 2011). Thus SOD acts as a pro-oxidant enzyme (Lortz et al. 2005; Lushchak et al. 2005), whenever the conversion of H_2_O_2_ into the HO^·^ radical is fostered in a cell type like the beta cell with its low abundance of H_2_O_2_-eliminating enzymes (Lenzen 2017). The hydrophilic O_2_^·−^ as a precursor of the hydrophilic H_2_O_2_ is a pro-oxidant, which as an anion is restricted in its mobility to the compartment of generation. In contrast to the negatively charged O_2_^·−^, the mobility of the electroneutral H_2_O_2_ molecule is virtually not restricted and thus reaches more sites than O_2_^·−^ for mediating toxicity.

## The special situation in the pancreatic beta cell ER

The ER in secretory cells such as the pancreatic beta cells is a subcellular compartment in which a very oxidized state prevails. The strongly oxidative milieu in the ER is the result of high amounts of H_2_O_2_ generated as well as of the low GSH/GSSG ratio (Appenzeller-Herzog 2011). This is achieved in an oxidation step mediated by PDI oxidoreductases (protein disulfide isomerases) [for details, see Lenzen (2017)].The ER acts as a H_2_O_2_ store, keeping a greater amount of the generated H_2_O_2_ in the ER due to a limited efflux of H_2_O_2_ into the cytosolic space. Insulin biosynthesis is a process which takes place constantly in the pancreatic beta cells. Its rate is high in particular at increased blood glucose concentrations in the postprandial state. Further accelerated is the increased rate of insulin biosynthesis in the prediabetic phase during the development of the T2DM state due to the prevailing insulin resistance under these conditions. This goes along with an increased rate of insulin misfolding, to which other islet cell hormones in other islet cell types are not prone, thus further accelerating ER stress (Lenzen 2017). In the prediabetic state, the insulin resistance induced hyperinsulinemia can be compensated by increased rates of insulin biosynthesis until the compensatory mechanisms ultimately collapse along with a gradually decreasing insulin synthesizing capacity. This results in continuously decreasing plasma insulin levels in the circulation due to progressive development of beta cell dysfunction along with an open state of ER stress.

Insulin comprises the major portion of the proteins synthesized in the pancreatic beta cell. For each proinsulin molecule folded in the ER three molecules of H_2_O_2_ are generated (Lenzen 2017) giving rise to a concentration of H_2_O_2_ higher than in many other cell types. This high level of H_2_O_2_ is also due to the fact that the ER membrane is rather impermeable for H_2_O_2_ (Konno et al. 2015). This is advantageous, since a significant proportion of H_2_O_2_ generated in the ER during proinsulin folding is consumed again for PDI re-oxidation (Hudson et al. 2015; Lenzen 2017). At the same time, high H_2_O_2_ levels in the ER are a risk factor with potential for beta cell dysfunction, as documented by the fact that an increase of the low expression level of the antioxidative enzyme Prdx4 in rodent beta cells through overexpression goes along with enhanced glucose-induced insulin secretion due to increased proinsulin mRNA transcription and insulin content (Mehmeti et al. 2012, 2014). An increased expression of the peroxiredoxin Prdx4 (Mehmeti et al. 2014, 2012) and the glutathione peroxidases GPx7 and GPx8 (Mehmeti et al. 2017) resident in the ER of many cell types can also improve the antioxidative capacity in the ER of rodent beta cells, though GPx7 and GPx8 are not expressed constitutively in their ER (Mehmeti et al. 2017). At variance from Prdx4, however, expression of these glutathione peroxidases in the ER has no positive effect on proinsulin folding (Lenzen 2017; Mehmeti et al. 2017).

Such a reinforcement of the antioxidative and functional capacity of the ER is thus an option to improve the resistance of the pancreatic beta cell against the challenges of diabetic metabolic stress. Exactly, this is the option which has been exercised by the ER in the human beta cell. This is likely the best possible choice of the human beta cell to protect the ER thereby allowing the human beta cell to remain viable over the long lifespan of the human being. Nevertheless, a Westernized lifestyle that is often associated with insufficient exercise and overweight remains a constant challenge also for a well-protected ER. Even the best protected proinsulin-folding machinery in the human ER can be overwhelmed through a lifestyle, which overcharges the functional capacity of the beta cell.

Though the rough ER compartment is relatively large, comprising one seventh of the beta cell volume (Dean 1973), the H_2_O_2_ generation during protein oxidative folding and its consumption during PDI re-oxidation takes place around the proinsulin molecule. The ER as a whole is occupied by the proinsulin-folding apparatus and rather oxidized through limited H_2_O_2_ transition into the surrounding cytosol. Due to this, there is no need for a long-distance travel of H_2_O_2_ within the ER.

## ER stress under type 1 and type 2 diabetic conditions: a comparison

When glucose tolerance starts to deteriorate towards the end of the prediabetic phase along with the start of a shrinking of the beta cell mass in the pancreas the pressure on the remaining beta cells through metabolic stress increases steadily. This poses an increased functional demand on the ER in each remaining beta cell, both in the quickly decreasing beta cell mass under T1DM conditions as well as in the gradually decreasing beta cell mass with its continuously increasing dysfunction under T2DM conditions (Lenzen 2017). In particular in the type 2 scenario, this ER stress can induce a process lasting many years or even decades, comprising both an initial phase of compensatory hyperinsulinemia due to insulin resistance and a subsequent phase of a slowly developing hypoinsulinemia due to deterioration of the insulin biosynthetic capacity in the beta cell ER and the gradual decease of the beta cell volume (Lenzen 2017).

This is a scenario which overstresses the remaining beta cells, which are still capable of providing insulin in the diabetic metabolic state and thus gives rise also to more misfolded proinsulin (Lenzen 2017). Therefore, the induction of a beta cell rest through provision of insulin by exogenous administration or by a blockade of insulin release by administration of a K_ATP_ channel opener such as diazoxide (Lenzen 2017) is a feasible measure. The desired result of this resting state of the insulin biosynthesis apparatus can be ideally a cessation of proinsulin folding and thus a reduction of H_2_O_2_ generation, thereby reducing the ER stress.

## Conclusions

**Fig. 1:**
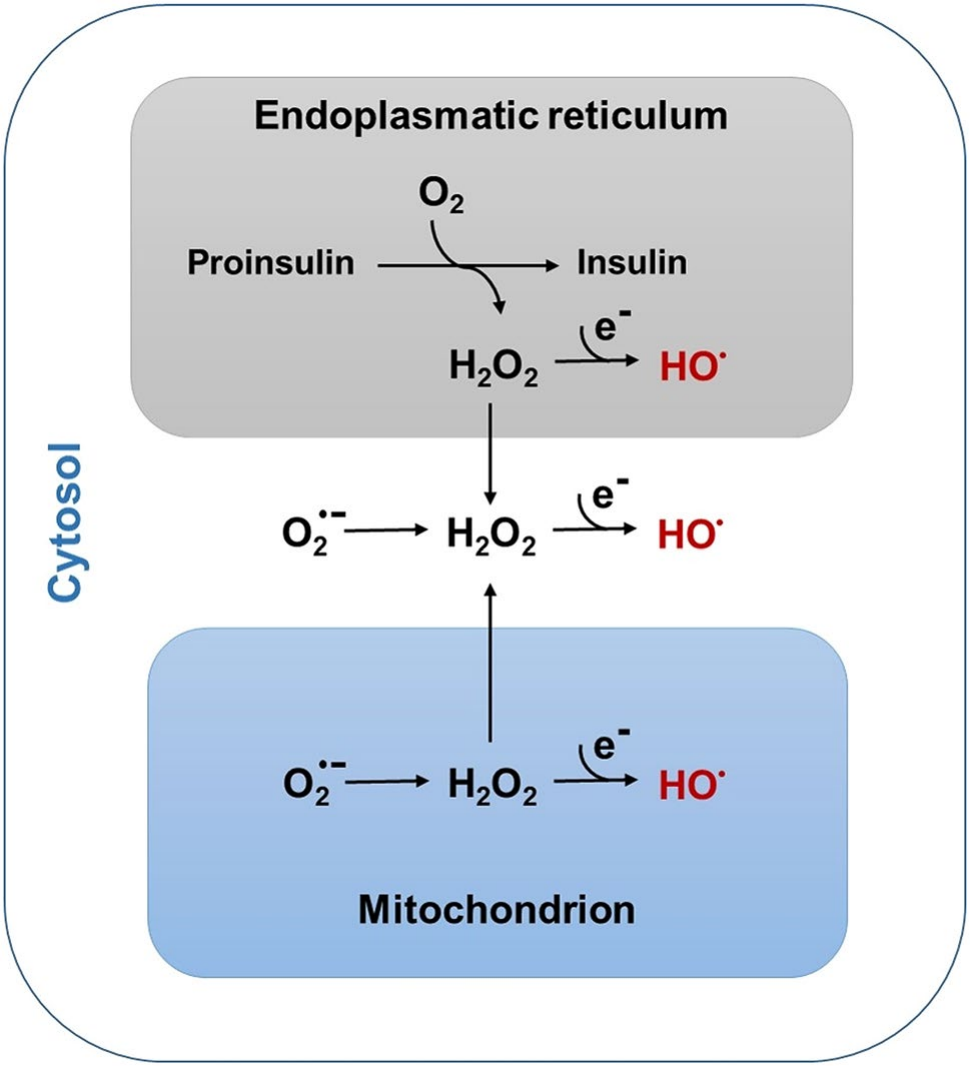
Formation of HO^·^ radicals as part of the homeostasis of reactive oxygen species in pancreatic beta cells. This figure depicts the different pathways of hydrogen (H_2_O_2_) generation in the different subcellular organelles, with H_2_O_2_ either originating from the superoxide radical in the cytosol and in the mitochondria or directly during proinsulin-folding in the endoplasmic reticulum (ER). The figure provides no quantitative information on the amounts of H_2_O_2_ formation in the different subcellular compartments

The pancreatic beta cell is characterized by a number of unique features (Lenzen 2021). One of these is the extreme vulnerability to ROS toxicity due to its weak antioxidative defense equipment compared to many other better protected cell types (Lenzen 2008, 2017; Lenzen et al. 1996; Tiedge et al. 1997). This is particularly true in states of diabetic metabolic stress. A crucial element of the beta cell demise in the developing diabetic state is the extreme toxicity of the HO^·^ radical. All biological structures in the beta cell are potential targets for this toxic action. In contrast, H_2_O_2_ causes only limited cellular dysfunction. However, due to its longevity and capability to reach all sites in the cell, H_2_O_2_ acts as a spreader for the toxicity of the HO^·^ radical reaching virtually all sites of the cell, where H_2_O_2_ in the presence of “free iron” can generate the HO^·^ radical (Fig. 1). This allows HO^·^ to execute its extremely toxic action leading to pancreatic beta cell dysfunction and ultimately to beta cell death in the vulnerable beta cell with its low enzymatic capacity for H_2_O_2_ inactivation as firmly documented (Grankvist et al. 1981; Lenzen 2008, 2017; Lenzen et al. 1996; Tiedge et al. 1997).

## Data Availability

Data for this review article originate from the publications in the reference list.

## Abbreviations

GPx: Glutathione peroxidase
H_2_O_2_: Hydrogen peroxide
O_2_^·−^: Superoxide radical
HO^·^: Hydroxyl radical
Prx: Peroxiredoxin
SOD: Superoxide dismutase
